# Emotional intelligence in action: theoretical models for educators to enhance learning and connection in the classroom: a conceptual review

**DOI:** 10.3389/fpsyg.2025.1660296

**Published:** 2025-10-22

**Authors:** Mokgata Alleen Matjie

**Affiliations:** Department of Business Management: HRM Program, University of Limpopo, Polokwane, South Africa

**Keywords:** emotional intelligence (EI), gardner’s multiple intelligences theory, the VARK model, academic succes, educators, learners/students, learning styles, classical conditioning

## Abstract

Emotional quotient (EQ) and emotional intelligence (EI) are often conflated with cognitive intelligence (CI); however, it distinctly refers to the quantifiable assessment of an individual’s emotional competencies and capabilities. A higher EQ is typically indicative of greater emotional proficiency, which is essential for various professionals, including educators, so that they can effectively tap into the CI of learners when teaching. Effective teaching transcends mere knowledge transfer; it necessitates the ability to connect emotionally with learners to foster an engaging and supportive educational environment. Unfortunately, many educators may not be cognizant of types of intelligences and learning styles and what these skills can contribute to their teaching efficacy, more specifically the emotional intelligence (EI). A successful educational interaction demands qualities such as compassion and patience, which help bridge the emotional gap between educators and learners. Thus, the cultivation of EI in educators can significantly enhance their ability to connect with diverse learners using different learning styles and intelligences, as well as other relevant theories. Professional development programs that focus on upskilling educators on the learning styles and intelligences, especially the EI and CI, can empower educators, equipping them with the necessary tools to leverage their emotional skills in the classroom for improved learning outcomes.

## Introduction

Since the publication of Daniel Goleman’s seminal work in 1995 (Goleman, 1995), emotional intelligence (EI or EI) has emerged as a pivotal area of study across diverse academic disciplines worldwide. Emotional intelligence, as defined by Salovey and Mayer (1990), constitutes a facet of social intelligence, encapsulating the ability to monitor one’s own emotions as well as those of others. This involves discriminating among various feelings and utilizing this information to guide thought processes and actions. In essence, EI is the capacity to perceive, assimilate, comprehend, and regulate emotions both intra- and inter-personally (Mayer and Salovey, 1997; Matjie, 2025).

Individuals with high emotional intelligence are often able to self-motivate to engage in a range of tasks, including those that are particularly challenging, provided they possess the necessary cognitive intelligence (CI). However, it is important to recognize that emotional intelligence is not solely determined by cognitive intelligence. Bar-On’s (1997) theoretical framework emphasizes this distinction by framing EI as a collection of non-cognitive competencies and skills that significantly impact one’s ability to navigate environmental demands and pressures. Consequently, in educational contexts, both EI and CI are essential for achieving academic success. While a learner’s EI equips them to effectively manage contextual challenges, CI provides the cognitive foundation required for excelling in intellectual tasks.

In advance of delving into the intricate complexities of emotional intelligence, it is imperative to consider the broader concept of “intelligence” and its implications within the educational paradigm. Intelligence, in its multifaceted nature, encompasses an array of dimensions intricately connected to cognitive functions such as memory, analytical reasoning, and problem-solving skills. A nuanced exploration of these diverse intelligences enriches our comprehension of human cognition and highlights the critical role of emotional intelligence as an integral aspect of comprehensive intellectual functioning.

## Intelligence and academic success

Intelligence is delineated as a general mental capacity encompassing reasoning, problem-solving, and learning (Colom et al., 2010). It represents a multifaceted construct that can be articulated both as a characteristic of human behavior and as an aggregation of cognitive abilities (Goldman and Pellegrino, 2013; Howard, 1993). Intelligence is frequently evaluated through standardized measures, most notably the intelligence quotient (CI) test, which functions as a quantifiable index of cognitive performance (Brody, 1999; Demetriou and Spanoudis, 2017). These assessments are expressly designed to gauge individuals’ capacities to acquire, comprehend, and apply knowledge, thereby offering a systematic approach to understanding intellectual capabilities (Brody, 1999).

A salient feature of intelligence is its temporal dynamism; it is subject to variation throughout an individual’s lifespan, with empirical evidence indicating a gradual decline in cognitive abilities as one ages (Alipour et al., 2024; Colom et al., 2010; Deary et al., 2007). This highlights the imperative of continual intellectual engagement and lifelong learning, suggesting that the maintenance of cognitive acuity is closely intertwined with sustained exposure to educational experiences (Zhi et al., 2024).

The relationship between intelligence and educational performance is well documented, with intelligence significantly influencing learners’ ability to perform academically (Brody, 1999; Deary et al., 2007; Lozano-Blasco et al., 2022; Sánchez-Álvarez et al., 2020). Given this interconnection, educators must acquire a nuanced understanding of the various dimensions of intelligence (Ayeni et al., 2024; Nachiappan et al., 2014; Zhi et al., 2024). By doing so, they can develop pedagogical strategies that are not only informed by theoretical knowledge but also tailored to the diverse cognitive profiles of their learners. This understanding can empower educators to create inclusive learning environments that effectively harness the diverse forms of intelligence, ultimately enhancing both teaching methodologies and learning outcomes (Calik and Birgili, 2013; Felder and Brent, 2005).

## Relations between the intelligences

There are different types of intelligence in education that educators should understand if they are to ensure the transfer of knowledge to learners effectively, namely, physical intelligence (PI), cognitive intelligence (CI), emotional intelligence (EI), and spiritual intelligence (SI) (Nachiappan et al., 2014), as defined and described in Table 1.

**Table 1:** Relations between types of intelligence.

Intelligence type	Definition	Key attributes	Core focus
Physical Intelligence (PI)	The ability to effectively manage and listen to one’s body.	Body awareness, energy management, health habits, and coordination	Health, stamina, and presence
Cognitive Intelligence (CI)	The capacity to think, learn, reason, and solve problems.	Logic, memory, analysis, and knowledge acquisition	Thinking and problem-solving
Emotional Intelligence (EI)	The ability to recognise, understand, and manage emotions in oneself and others.	Empathy, emotional regulation, and social skills	Relationships and self-awareness
Spiritual Intelligence (SI)	The ability to apply meaning, values, and a sense of purpose to one’s life and work.	Meaning-making, purpose, integrity, compassion	Vision, ethics, and life fulfilment

It is thus crucial to remember that EI alone cannot enhance academic achievement, hence the introduction of other intelligences in this conceptual review, despite the title being about EI alone. As shown in Table 1, physical intelligence (PI) refers to the ability to effectively manage and tune in to the body’s signals and needs (Neal and Harpham, 2012). This multifaceted construct encompasses several key components, including: body awareness the ability to recognize and interpret bodily sensations and movements, which facilitates better physical responses and enhances overall well-being; energy management the skill to regulate one’s energy levels throughout the day, ensuring optimal performance and preventing fatigue; health habits the adoption and maintenance of behaviors that promote physical well-being, including nutrition, exercise, and rest; and coordination the capacity to harmonize muscle movements for efficient and purposeful action, thereby improving physical skills and reducing the risk of injury (Nachiappan et al., 2014). PI is the foundation for all other intelligences (Wigglesworth, 2012).

Cognitive intelligence, commonly referred to as rational or intelligence quotient (CI), encompasses the ability to engage in complex thought processes such as reasoning, learning, problem-solving, and analytical thinking (Ronthy, 2014). It involves various cognitive functions, including logical reasoning, memory retention, analytical skills, and the acquisition of knowledge (Nachiappan et al., 2014; Zohar and Marshall, 2004; Wigglesworth, 2012).

Emotional intelligence represents a critical competency involving the ability to recognize, comprehend, and regulate emotions, both within oneself and in interpersonal contexts (Salovey and Mayer, 1990). It encompasses several key components, including empathy, emotional regulation, and social skills, which collectively facilitate effective communication, conflict resolution, and nurturing of relationships (Nachiappan et al., 2014). Research studies have demonstrated how EI and CI differ. While EI can be learned and acquired as a skill, CI is largely predetermined (Bar-On, 1997; Goleman, 1996; Goleman et al., 2013; Zohar and Marshall, 2004).

Spiritual intelligence (SI) can be defined as the capacity to derive meaning, uphold values, and cultivate a sense of purpose within both personal and professional realms (Covey, 2005; Däderman et al., 2013). It encompasses several key dimensions, including meaning-making, purpose-driven behavior, integrity, and compassion (Däderman et al., 2013; Nachiappan et al., 2014). SI is considered the ultimate intelligence (Churchill, Gandhi, and Mandela) (Zohar, 2005) and the foundation of both CI and EI. SI is characterized by wisdom and peace in the face of chaos (Wigglesworth, 2013). Spiritual intelligence leads to more emotional intelligence; that is, emotional intelligence strengthens spiritual intelligence (Pinto et al., 2024). In addition to strong CI and EI, she argues, a strong foundation in Physical Intelligence (PI) and a willingness to develop Spiritual Intelligence (SI) are needed. When these four come together, she writes, the result is Deep Intelligence (Wigglesworth, 2014).

### The question remains

Does any or all of the above intelligences lead to academic performance for learners? The answer to this question might be found in the section below.

## Various intelligences and their relations to academic performance

The investigation into the various types of intelligence is critical for understanding their relationship with academic performance. As outlined in the previous discussion, it is essential to identify the specific type of intelligence that correlates most strongly with academic success. This identification can inform the training of educators, equipping them with strategies to effectively engage and nurture that intelligence in learners. According to Zhao (2017), academic achievement is defined as the measurable performance of learners in mastering academic knowledge and skills, assessed through examinations following a systematic process of learning. This performance not only reflects a student’s understanding but also signifies the extent to which they have internalized the requisite knowledge and competencies (Liang et al., 2020). Therefore, understanding the interplay between different intelligences and academic achievement is pivotal in developing effective educational practices (Grass et al., 2017; Shi and Qu, 2022).

Physical intelligence encompasses health, stamina, and presence, underscoring the importance of a holistic approach to physical wellness and body awareness in various aspects of life (Ronthy, 2014; Wigglesworth, 2012). Physical fitness can be considered a good measure of the body’s capacity for exercise and also provides an important indicator of health (Real-Pérez et al., 2022). Some studies concluded that physical intelligence does not have positive effects on learners’ academic performance (Bakir, 2024; Gil-Espinosa et al., 2020; Strong et al., 2005). Contrarily, some studies found that being physically healthy, fit, or strong does relate to academic performance (Du Toit et al., 2011; Gil-Espinosa et al., 2020; Hillman et al., 2009; Li and Zhang, 2022; Real-Pérez et al., 2022), thus making PI crucial for learners if they are to perform academically. CI is a key factor that can be consistently used to predict academic achievement (Grass et al., 2017; Liu et al., 2021; Merriam, 2004; Miriam et al., 2011; Stadler et al., 2016; Shi and Qu, 2021, 2022). When it comes to EI, studies found that EI plays a vital role in personal development and social interactions, influencing overall well-being and professional success (MacCann et al., 2020). MacCann et al. (2020) further propose that EI should be incorporated into the academic curriculum and that learners should be encouraged to build social relationships at school to unlock aspects of their EI that may enhance their success in the social sciences. These findings are corroborated by Quílez-Robres et al. (2023), who propose that schools should establish programs to stimulate emotional intelligence at the school level to improve learners’ personal development and academic performance (Perera and DiGiacomo, 2013; Petrides et al., 2004). Moreover, educators’ training should include modules on EI to enable them to nurture emotional competencies in learners (Amponsah et al., 2024). Specific EI domains such as self-perception, empathy, impulse control, and stress tolerance are particularly associated with academic performance (Farah-Franco et al., 2025). This suggests that EI is a vital component of academic performance and should be integrated into the curriculum while teachers are trained to be emotionally competent (Zhou et al., 2024). Despite that, EI alone seems not viable to ensure ultimate academic performance; hence, further studies revealed that SI plays a crucial role in individual well-being and development (Däderman et al., 2013; Nachiappan et al., 2014). According to Midi et al. (2019), Rahimi (2017), and Zhou et al. (2024), spiritual intelligence predicts educational achievement in both university and school contexts, making it imperative for schools to implement programs that foster students’ spiritual intelligence.

In conclusion, different intelligences are strong predictors of academic performance; however, results vary depending on the type of intelligence measured, the theoretical model used, and the cultural context (Lozano-Blasco et al., 2022). Based on the above discussion, the question arises: What training programs should be developed for educators to integrate all forms of intelligence (not EI only) into teaching methods and learning styles? Before addressing this, the theoretical framework that underpins this study must be examined.

## Theoretical framework

To comprehend the role of intelligences on academic performance, more specifically, the role of EI to align with the title of the paper, we identified the following theories, namely, knowledge processing theory, classical conditioning theory and Gardner’s Theory of Multiple Intelligences to evaluate if cognitive abilities resulting from high EI can alone lead to academic success, or students can be primed to become academically successful without any intelligences or lastly to ascertain if other intelligences should be applied to bolster EI leading to academic success.

### Knowledge process theory

According to the knowledge process theory, the acquisition of knowledge and the learning process are profoundly intertwined with the cognitive capabilities of learners (Deary et al., 2007). Learners endowed with higher cognitive abilities encompassing not only Intelligence Quotient (IQ) but also Emotional Quotient (EQ) tend to excel in swiftly and accurately converting essential information into lasting memory. This cognitive agility allows their brains to produce increasingly effective and organized information, thereby significantly boosting academic performance (Wang and Liu, 2000). This theory implies that EI alone cannot enhance learning, but a combination with IQ can make a valuable difference in a learner’s academic achievements.

In contrast, learners with lower cognitive abilities may miss out on critical knowledge during their educational journey. This gap in understanding can lead to a diminished capacity for effective information output, ultimately resulting in poorer academic outcomes (Miriam et al., 2011). Importantly, IQ should not be viewed in isolation. Instead, a comprehensive approach that harmonizes Spiritual Quotient (SQ), Emotional Quotient (EQ), and Physical Quotient (PQ) is essential to maximizing a student’s overall cognitive potential. This holistic balance enhances the ability to encode and apply relevant information, an essential factor in academic success.

Educators and educational institutions must remain vigilant in maintaining this complex equilibrium. Empirical studies have provided compelling evidence that although each form of intelligence can independently contribute to academic performance (Amponsah et al., 2024; Farah-Franco et al., 2025; Gil-Espinosa et al., 2020; Li and Zhang, 2022; Zhou et al., 2024), the CI amplifies the benefits of these intelligences (Demetriou and Spanoudis, 2017; Liu et al., 2021).

### Classical conditioning theory

Classical conditioning, first demonstrated through the experiments of Ivan Pavlov, remains a cornerstone of psychological learning theory, highlighting the importance of associative learning (Pavlov, 1913; Watson, 1924). This process involves forming connections between a neutral stimulus originally unrelated to any specific response and an unconditioned stimulus that naturally evokes a reaction (Eelen, 2018; Totani et al., 2019; Watson, 1924). With repeated pairings, the neutral stimulus comes to elicit a similar response, thus illustrating how both animals and humans learn through association (Amd et al., 2019; Pavlov, 1913; Rehman et al., 2025; Watson, 1924). In the educational setting, classical conditioning underscores how learners interact with their environment. When classrooms incorporate enjoyable and stimulating experiences (the unconditioned stimulus), students begin to associate these positive emotions with learning itself. This leads to enthusiasm and intrinsic motivation, key outcomes of the conditioned response. Educators who emphasize meaningful engagement can therefore foster an atmosphere that enhances students’ emotional connection to learning, allowing them to thrive beyond traditional reward-based systems.

### Gardner’s theory of multiple intelligences

Howard Gardner’s theory of multiple intelligences offers a comprehensive and inclusive perspective on human intellectual potential that transcends the limits of traditional CI testing (Gardner, 1983; Gouws, 2007; Yang, 2013). Gardner posits that intelligence involves the ability to solve problems or create products of value using diverse methods, highlighting the plurality of cognitive strengths among individuals (Gardner, 1983).

This model carries significant implications for vocational and general education, advocating for learner-centered approaches and pedagogical strategies tailored to individual strengths and preferences (Sadiku et al., 2020; Sener and Cokcaliskan, 2018; Yavich and Rotnitsky, 2020). Gardner’s work also lays the foundation for multidimensional assessment strategies that reflect a broader array of human capabilities (Gardner, 1983, 1999). Furthermore, the theory promotes entrepreneurial and creative thinking, encouraging learners to approach challenges with flexibility and innovation (Calik and Birgili, 2013; Yang, 2013).

The three theories discussed play a pivotal role in the development of theoretical models aimed at educators, facilitating the enhancement of learners’ emotional intelligence (EI). By integrating these theories into educational practices, educators can foster an environment that nurtures emotional growth and responsiveness among learners. Understanding the underlying principles of these theories allows for the creation of effective strategies and resources tailored to bolster EI in various learning contexts.

## Theoretical models for practical implementation by educators

Based on the aforementioned information and the theories discussed, we have developed a set of theoretical models for educators to implement in their classrooms aimed at fostering emotional intelligence (EI) about learners’ achievements. These tools are designed to enhance student engagement, promote self-awareness, and cultivate interpersonal skills, thereby supporting the holistic development of each learner. Throughout this initiative, we emphasize the importance of integrating EI practices into the educational framework to ultimately improve academic outcomes and personal growth.

### Theoretical model 1: a holistic intelligence framework leading to academic success

**Figure 1:**
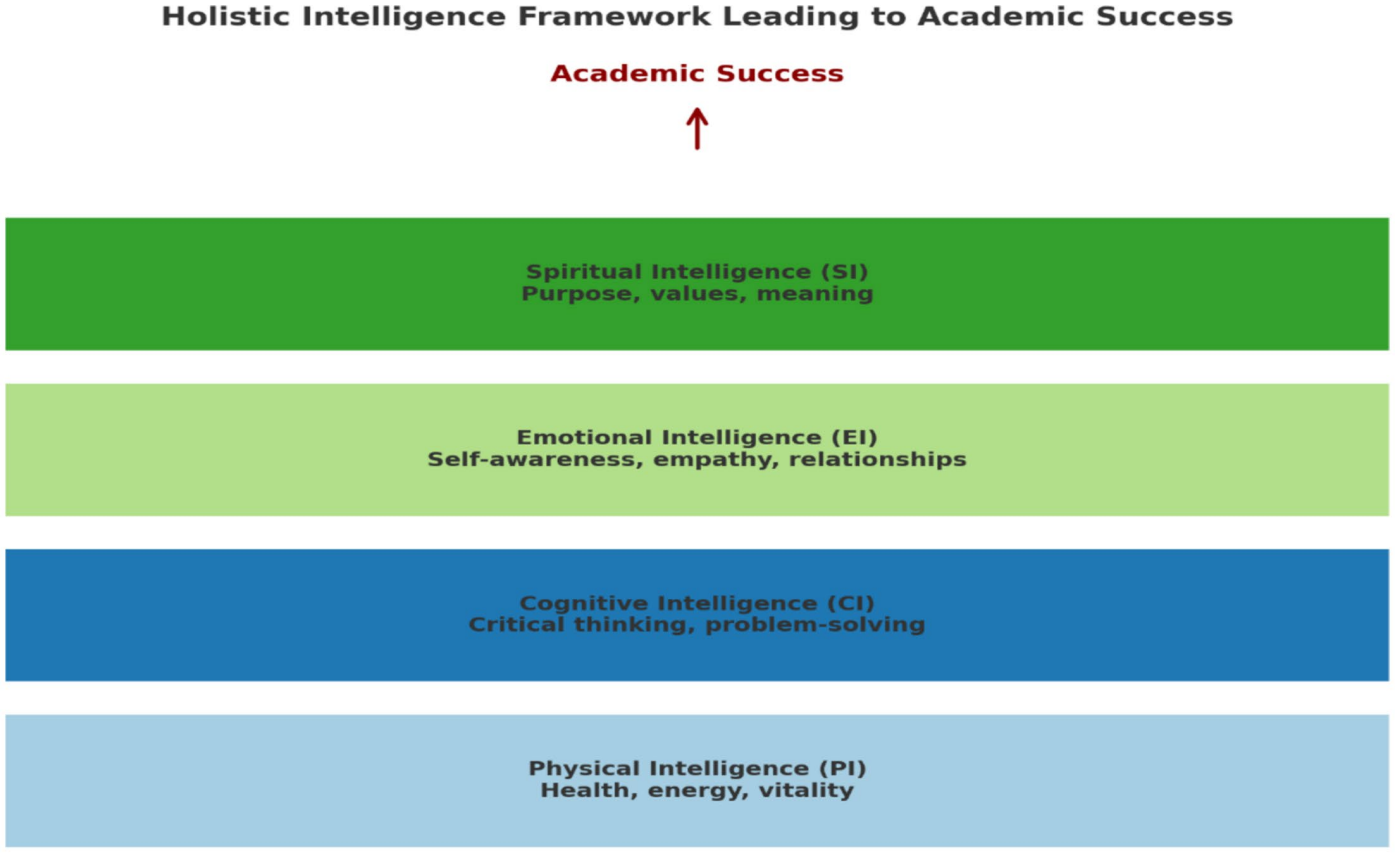
A holistic intelligence framework leading to academic success.

It is evident from the above discussion that spiritual intelligence provides meaning, existence, reasons and guidance for learners, hence it is at the apex of the model (Figure 1).

The proposed framework positions physical (PI), cognitive (CI), emotional (EI), and spiritual intelligence (SI) as layered domains that collectively underpin academic success. This model advances a multidimensional view of education, challenging traditional paradigms that privilege cognitive ability as the primary determinant of achievement. A critical analysis of this framework reveals both its strengths and limitations in theory and practice.

Placing PI at the base of the framework is conceptually sound. Research demonstrates that physical health and energy management significantly affect students’ attention, memory, and engagement (Donnelly et al., 2016). Sleep, nutrition, and physical activity have direct implications for academic performance, positioning PI as a legitimate foundation. However, the model risks reductionism if it implies that PI functions solely as a prerequisite. Empirical studies suggest a reciprocal relationship, where cognitive engagement also motivates healthier behaviours, indicating that PI and CI interact dynamically rather than hierarchically (Ratey, 2008). The framework highlights CI as the intellectual driver of learning, echoing long-standing traditions in educational psychology that emphasise problem-solving, reasoning, and critical thinking (Neisser et al., 1996). While CI remains essential, privileging it as the core perpetuates the limitations of IQ-based models. Such approaches have been critiqued for underestimating the importance of social, emotional, and cultural dimensions of learning (Gardner, 2011). A narrow focus on CI risks reinforcing inequities by valorising test-based achievement while neglecting broader competencies. The integration of EI acknowledges that learning is inherently social and emotional. Students with strong EI demonstrate improved peer relationships, stress management, and resilience, indirectly supporting academic outcomes (Mayer et al., 2016). This dimension enhances the framework’s relevance for modern educational contexts that value collaborative and inclusive learning. Nonetheless, questions remain about the measurability and cultural transferability of EI (Zeidner et al., 2012). Without accounting for cultural variability, EI may risk being reduced to a Western-centric construct. At the apex, SI reflects the argument that education must move beyond functional skills to cultivate values, purpose, and moral reasoning (Zohar and Marshall, 2000a, 2000b). This resonates with holistic education philosophies and aligns with contemporary calls for purpose-driven learning. However, SI remains a contested concept, particularly in secular and pluralistic settings, where spiritual discourse may inadvertently privilege certain worldviews. To ensure inclusivity, SI must be operationalised broadly, encompassing existential reflection, ethical reasoning, and a search for meaning rather than specific religious traditions (Vaughan, 2002). The framework presents academic success as a linear culmination of the four intelligences. While visually appealing, this trajectory oversimplifies the complex and reciprocal relationships between the domains. For instance, academic success can reinforce EI through enhanced self-efficacy and SI through a heightened sense of purpose. Thus, the relationship is better understood as cyclical rather than unidirectional.

The holistic intelligence framework presents several strengths: it integrates diverse forms of intelligence, challenges reductionist IQ models, and offers practical entry points for educators through wellness programs, emotional literacy training, and values-based curricula. However, it also has limitations: its hierarchical presentation oversimplifies the interdependencies among different intelligences, social intelligence (SI) remains pedagogically challenging, and it does not sufficiently address socio-cultural factors such as poverty, systemic inequality, or institutional barriers that significantly influence academic outcomes (OECD, 2019).

While the framework serves as a valuable corrective to IQ-centric models of student achievement by recognizing multiple dimensions of human development, a critical perspective highlights the need for greater nuance. Rather than viewing these intelligences as a rigid hierarchy, future adaptations should conceptualize them as an interdependent ecosystem, where physical, cognitive, emotional, and spiritual dimensions continuously interact. This reframing would not only capture the complexity of learning but also enhance the framework’s applicability across diverse cultural and educational contexts. These interconnected forms of intelligence illustrate the holistic nature of human development and emphasize the necessity for a balanced approach to personal growth and self-actualization, which educators and learners should be prepared to embrace (Liu et al., 2021; Lozano-Blasco et al., 2022; Nachiappan et al., 2014). It is recommended that this theoretical model be piloted in various educational contexts across different countries, utilizing practical implementation examples. This approach will allow for the examination of cultural limitations and their impact on the model’s efficacy (El-Saftawy et al., 2024), providing valuable insights into its potential for successful integration in distinct educational systems.

#### Practical implementation of the framework

1. Spiritual intelligence (SQSI): cultivating meaning and purpose

In education, spiritual intelligence supports students in connecting their studies and life experiences to a deeper sense of purpose.

- Implementation

      ◯  Reflective journaling assignments where students link academic learning to personal values.
      ◯  Service-learning projects (e.g., sustainability initiatives, volunteering) that allow students to experience interconnectedness with their community and environment.
      ◯  Classroom discussions around ethics, responsibility, and moral dilemmas in real-world case studies.

2. Emotional intelligence (EQEI): building self-awareness and empathy

Emotional intelligence equips students to manage their emotions, navigate peer relationships, and foster collaborative learning environments.

- Implementation:

      ◯  Group projects with structured peer-feedback to practice empathy, conflict resolution, and collaboration.
      ◯  Role-play activities and classroom dialogues that help students recognize and respond to different emotional perspectives.
      ◯  Mentorship or peer-support systems that promote emotional resilience and a sense of belonging.

3. Cognitive intelligence (IQCI): strengthening critical and analytical thinking

Cognitive intelligence ensures students can solve problems, think critically, and apply knowledge across disciplines.

- Implementation:

      ◯  Integrating case-study analysis and debate sessions to foster evidence-based reasoning.
      ◯  Encouraging research-based projects where students apply theoretical concepts to practical challenges.
      ◯  Embedding problem-based learning (PBL) in curricula to simulate real-world scenarios requiring critical thinking.

4. Physical intelligence (PQPI): supporting vitality and optimal performance

Physical intelligence provides the foundation for sustained focus, energy, and engagement in learning.

- Implementation:

      ◯  Classroom movement breaks, mindfulness stretches, or short physical activities during lessons to maintain energy levels.
      ◯  Workshops on nutrition, sleep, and stress management tailored to student lifestyles.
      ◯  School or university wellness programs that integrate sports, fitness challenges, and ergonomic study practices.

### Theoretical model 2: combined integrated academic success and VARK model

**Figure 2:**
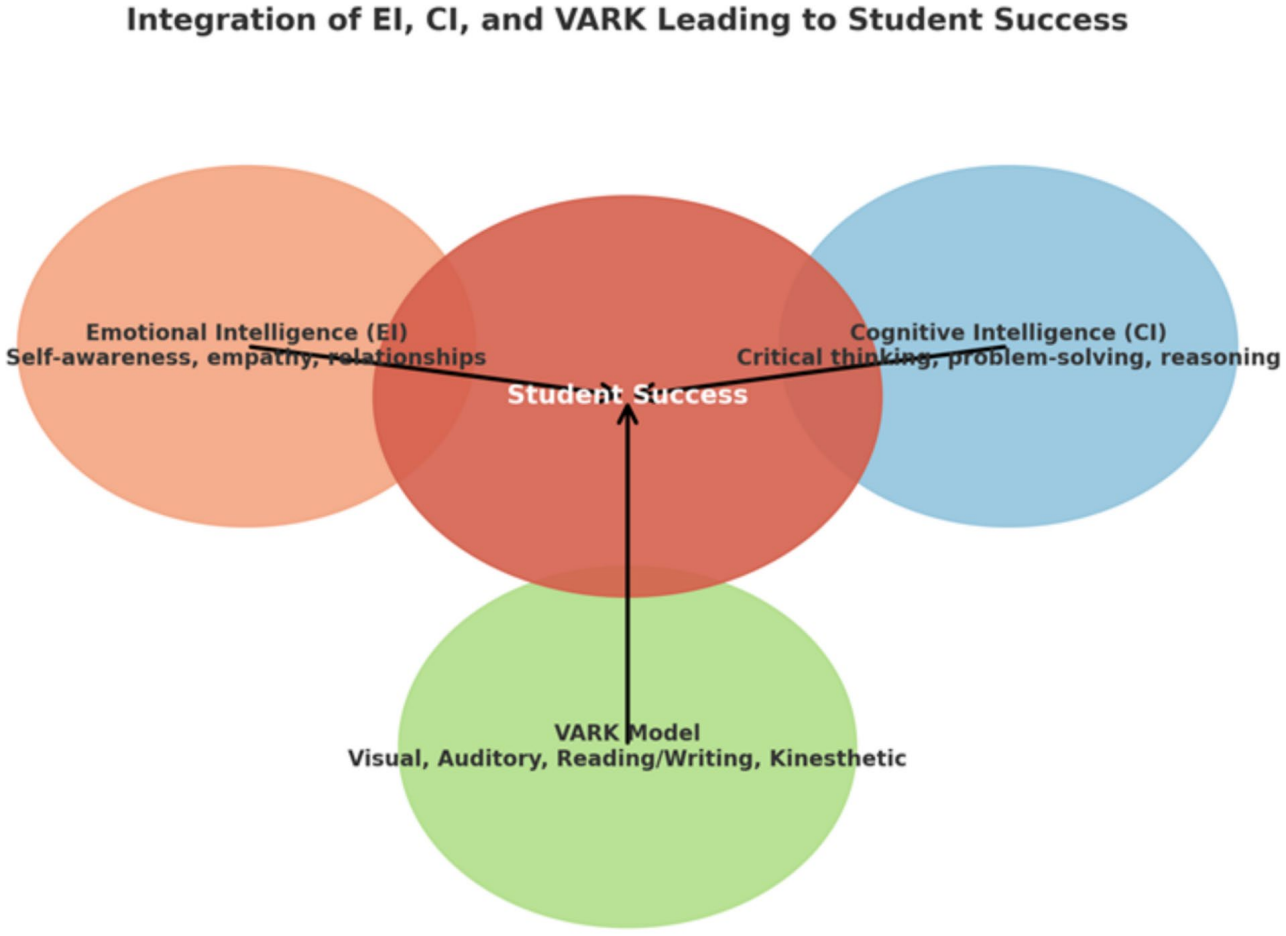
Combined integrated academic success and VARK model.

**Figure 3:**
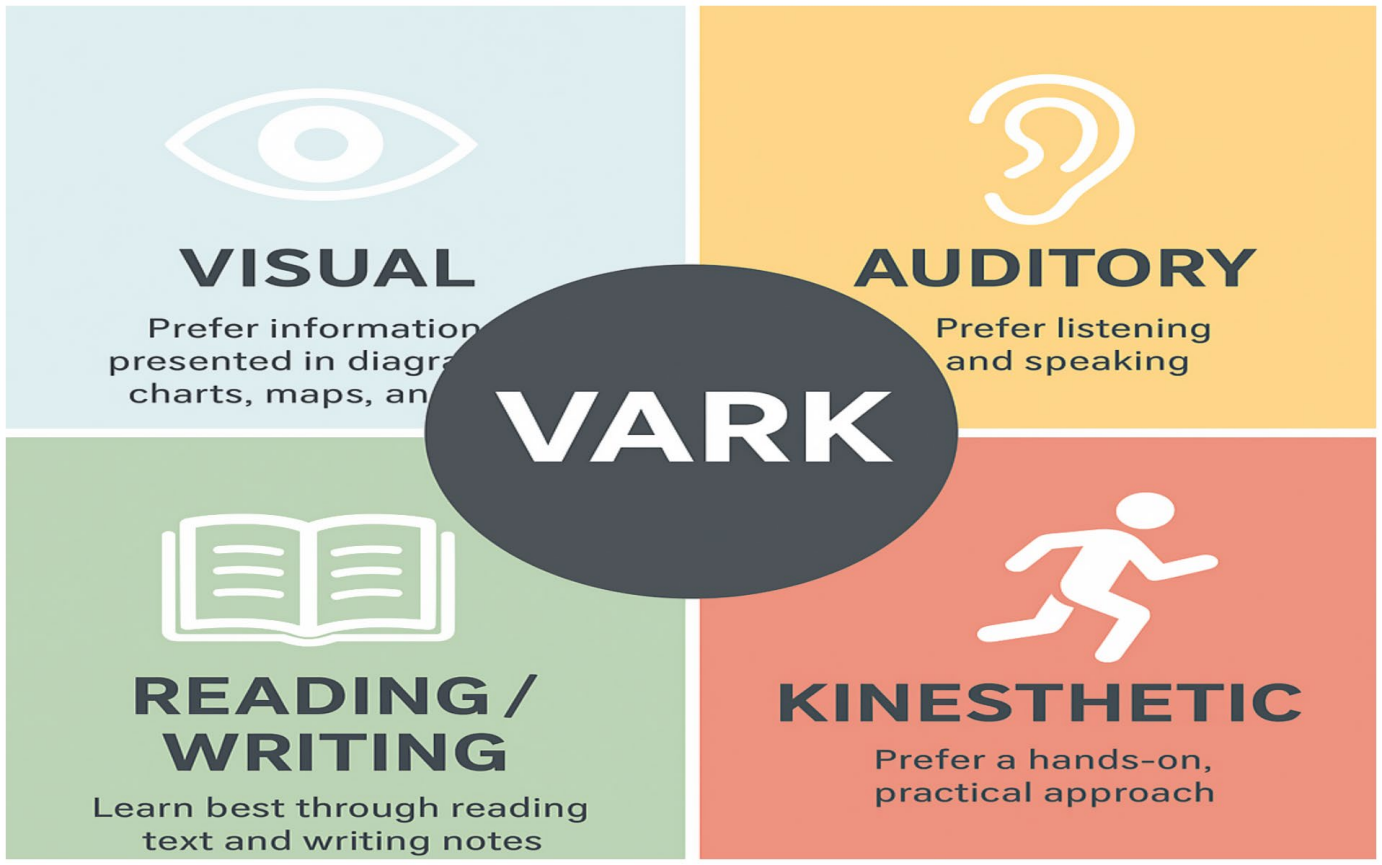
The VARK model.

The second pedagogical theoretical model (Figure 2) for educators is the integration of various intelligences, specifically emotional intelligence (EI) and cognitive intelligence (CI), alongside diverse learning styles as outlined in the VARK model. This combined approach aims to enhance academic performance by fostering a more holistic educational experience. The Combined Integrated Academic Success and VARK model prioritizes EI and CI while also taking into account the four distinct learning styles identified in the VARK model. This approach provides a nuanced perspective that can significantly benefit educators seeking to enhance both EI and CI in their learners, ultimately leading to improved academic achievements. The effective application and expression of emotional and cognitive intelligence are significantly influenced by the educational context. This influence is particularly evident through the utilization of suitable pedagogical strategies and the consideration of individual learning styles, as demonstrated by several studies (Liu et al., 2021; Stadler et al., 2016; Shi and Qu, 2021, 2022). This underscores the importance of a tailored approach to teaching and learning, promoting an environment where both emotional and cognitive intelligence can thrive. However, due to the introduction of learning styles as a means to effective learning and ultimate academic success, the Combined Integrated Academic Success and VARK model (see Figure 3).

The VARK model is a widely recognized framework that categorizes learners into four primary styles: Visual, Auditory, Reading/Writing, and Kinesthetic (Fleming, 2006; Kaushik and Joshi, 2016). Each category reflects distinct learning preferences and cognitive attributes:

- Visual learners favor the use of images, diagrams, and other visual aids, demonstrating a strong memory for colors, spatial layouts, and shapes (Gholami and Bagheri, 2013; Deshmukh et al., 2014).
- Auditory learners excel in environments that emphasize verbal instruction and group discussion, showing a preference for listening and interpersonal communication (Price and Griggs, 1985; Gholami and Bagheri, 2013).
- Reading/Writing learners thrive on text-based resources, often engaging in extensive reading and meticulous note-taking as part of their learning strategy (El-Saftawy et al., 2024; Gholami and Bagheri, 2013).
- Kinesthetic learners are inclined toward hands-on, experiential learning activities. They may struggle with passive lecture formats and benefit most from movement-based and collaborative tasks (Deshmukh et al., 2014; El-Saftawy et al., 2024).

Research indicates that emotional intelligence (EI) plays a crucial role in fostering self-discipline and intrinsic motivation among learners, both of which are critical components of academic success (Amponsah et al., 2024; Farah-Franco et al., 2025; Leasa et al., 2017; Singh et al., 2008; Zhou et al., 2024). Conversely, cognitive intelligence (CI) is vital for analyzing and synthesizing complex information, thereby enabling learners to engage in higher-order thinking processes (Ronthy, 2014). Learning styles are essential constructs that reflect the diverse preferences and strategies individuals employ to absorb, process, and retain information (Fleming, 2001, 2006). They are typically classified according to sensory preferences, providing valuable insights into how different learners engage with learning materials (Fleming, 2001; Othman and Amiruddin, 2010). By identifying and understanding these distinctive learning preferences, educators can tailor their instructional approaches to improve educational outcomes.

In conclusion, the second pedagogical theoretical model for educators emphasizes the integration of various intelligences, specifically emotional intelligence (EI) and cognitive intelligence (CI), along with diverse learning styles as outlined in the VARK model. This combined approach aims to enhance academic performance by creating a more holistic educational experience. Educators should utilize different learning styles to engage various learners and stimulate their cognitive and emotional abilities (Li and Xue, 2023). All learning styles aim to activate either CI or EI, or both. Therefore, a pilot program should be implemented to test the viability of this model while considering different cultural contexts, as suggested by Dantas and Cunha (2020).

#### Practical applications of the integrated academic success model alongside the VARK model can enhance teaching efficacy

**Integrated application in education**

An effective classroom can integrate EI, CI, and VARK simultaneously:

- A case study discussion (CI) may start with group collaboration (EI) while providing multiple learning materials (VARK), a diagram for visual learners, oral explanations for auditory learners, reading guides for text-focused learners, and a hands-on role-play for kinesthetic learners.
- This integration ensures holistic development: students think critically, manage emotions, and learn in ways that suit their strengths.

The constructs of cognitive intelligence (CI) and emotional intelligence (EI), along with various learning styles, such as those delineated by the VARK model, are not universally applicable across different cultural contexts. Research indicates that these models are significantly shaped by varying cultural norms, educational methodologies, and societal values (El-Saftawy et al., 2024; Li and Xue, 2023). Specifically, while the VARK model categorizes learning preferences into visual, aural, read/write, and kinesthetic modalities, the manifestation and interpretation of these preferences can vary markedly among different cultural groups. Moreover, there is a lack of universal empirical evidence to support the notion that aligning teaching methods with specific learning styles consistently enhances educational outcomes (Dantas and Cunha, 2020). This underscores the importance of recognizing and adapting to the diverse educational needs that arise in multicultural settings.

### Theoretical model 3: Gardner’s (1983) multiple intelligence theory (MIT) (intelligence in psychology)

The usage of MIT in educational settings has been well documented and approved to work (Gouws, 2007). In his seminal work, Gardner initially identified seven distinct forms of intelligence: linguistic–verbal, logical-mathematical, musical, spatial, bodily-kinesthetic, interpersonal, and intrapersonal. As his research evolved, he introduced additional modalities, specifically spiritual intelligence (Gardner, 1999; Tirri and Nokelainen, 2008) and existential intelligence in the second edition of his book (Calik and Birgili, 2013). Numerous researchers assert that each type of intelligence is characterized by specific traits and abilities that delineate it from others (Barrington, 2004; Gardner, 1999; Sadiku et al., 2020; Sener and Cokcaliskan, 2018; Yavich and Rotnitsky, 2020), as illustrated in Table 2. Gardner’s (1983, 1999) theory suggests that everyone has different strengths and learning styles across these areas.

**Table 2:** Gardner’s multiple intelligences, learning styles and their characteristics.

Learning style	Relevant intelligence	Learning methods	Characteristics
Visual learners	Spatial	Prefer learning methods that combine visual aspects, such as presentations, pictures and others (Yavich and Rotnitsky, 2020)	Learners are influenced by educators’ body language and tend to prefer sitting at the front of the classroom (Sener and Cokcaliskan, 2018).
Auditory learners	Musical	Perceive the environment with the sense of hearing: music, sounds, words (Yavich and Rotnitsky, 2020)	The volume, frequency, and speed of speech significantly impact their learning. Research shows that auditory learners prefer reading in class, enhancing their engagement with the material (Sener and Cokcaliskan, 2018).
Verbal learners	Linguistic	Learn by verbalising words and writing (Yavich and Rotnitsky, 2020)	These learners actively engage with what they read and take notes while listening, enhancing their comprehension and retention of information (Sener and Cokcaliskan, 2018).
Intangible learners	Bodily kinesthetics	They prefer combining movements and tactile sensation, such as using hands (Yavich and Rotnitsky, 2020)	Learners gather information by interacting with the physical and motion world, needing hands-on engagement. They struggle with tasks requiring prolonged focus (Sener and Cokcaliskan, 2018).
Group learners	Interpersonal	Prefer group activities and learning through social interaction (Yavich and Rotnitsky, 2020)	Very good communication skills, both verbally and non-verbally. Leaners prefer to teach and guide others (Sener and Cokcaliskan, 2018).
Individual learners	Intrapersonal	Prefer self-study and are intrinsically motivated. They can gauge their learning efforts (Yavich and Rotnitsky, 2020)	Emotionally competent learners who can express their learning process and express personal feelings (Sener and Cokcaliskan, 2018).
Logical learners	Logical mathematical	They learn when making logical connections with the content (Yavich and Rotnitsky, 2020)	These learners can analyse different ways of thinking (Sener and Cokcaliskan, 2018).
Hands-on learners	Naturalist	Hands-on activities, outdoor explorations, and projects related to the natural world can be highly engaging and effective (Gardner, 1999; Sadiku et al., 2020)	Students prefer outside environments learning elements of nature like plants, animals, and weather patterns (biology, zoology, or environmental science) (Gardner, 1999; Sadiku et al., 2020)

## Learning styles for Gardner’s 8 multiple intelligences

To facilitate learning, we combined multiple intelligences with learning styles as well as methods of teaching to enable teaching as on Table 2.

MIT presents a thought-provoking perspective on intelligence, suggesting that individuals do not possess a fixed amount of intelligence from birth. Building on this premise, Gardner (1999) expanded the concept by developing various learning styles designed to enhance the multiple intelligences that learners exhibit. He defined learning styles as the unique ways in which learners perceive and process information during their educational experiences. Extensive research, as highlighted by Jena (2018), supports the idea that a diverse range of learning styles exists among individuals. This variety in learning preferences emphasizes the importance of teaching methods that educators can utilize to cater to different styles, enabling them to equip learners with essential skills applicable across all types of learning, regardless of their inclinations (Felder and Brent, 2005).

As learners gain insights into their learning styles, their capacity to absorb and retain information improves significantly. Additionally, the effectiveness of learning experiences can be enhanced when teaching methods align with learners’ preferred ways of learning. However, the overarching goal is not merely to customize educational experiences for each student individually, but rather to foster the development of versatile learning skills that are beneficial across all learning modalities (Felder and Brent, 2005). Educators should consider implementing practical applications of the integrated academic success model alongside the VARK model to stimulate learning in relation to MIT’s theoretical framework.

Nonetheless, caution is warranted when applying the Multiple Intelligences theory and the VARK models, as they have notable limitations. These limitations primarily revolve around a lack of strong empirical evidence and a tendency to oversimplify complex learning processes, which can lead to mislabeling students and neglecting a comprehensive, flexible approach to learning. Critics argue that neither framework has substantial scientific backing, and the idea of matching instruction to a single “intelligence” or “style” does not necessarily improve learning outcomes (Biscardi et al., 2019; Sood and Sarin, 2021).

The main limitations of Multiple Intelligences (MI) theory include a lack of empirical evidence and reliable assessment tools, confusion with learning styles, challenges in practical application—especially in large classrooms—and a potentially restrictive view of identity. Critics contend that the theory’s “intelligences” are often merely talents or skills rather than distinct cognitive systems, arguing that the brain functions more as interconnected networks than isolated modules (Calik and Birgili, 2013; Klein, 1997). As a result, caution should be taken when implementing this approach, and the usage of multiple approaches is encouraged.

### Practical application/implementation of Gardner’s (1983) multiple intelligences theory (MIT)

Howard Gardner’s Multiple Intelligences Theory (Gardner, 1983) challenges the traditional view of intelligence as a single ability. Instead, he identifies at least eight independent intelligences: linguistic, logical-mathematical, spatial, bodily-kinesthetic, musical, interpersonal, intrapersonal, and naturalistic. Some of its strengths include: broadened definition of intelligence, which includes talents in areas like music and art that traditional IQ tests often overlook (Gardner, 1983). The theory has a menainful impact on education because it advocates for student-centered teaching, encouraging diverse methods to engage different intelligences, such as storytelling (linguistic), hands-on experiments (bodily-kinesthetic), and visual aids (spatial) (Armstrong, 2017). It leads to motivation and engagement by valuing students’ unique strengths; the theory fosters self-confidence and a positive learning environment (Bas, 2016). And it can be applied widely, because it is relevant beyond education, influencing leadership, organizational learning, and career development (Shearer, 2018) (see Table 3).

**Table 3:** Gardner’s multiple intelligences and implementation examples.

Intelligence	Key strengths	Practical implementation examples (education/students)
1. Linguistic (word smart)	Language, reading, writing, storytelling	Essays, debates, poetry writing, role-plays, and student presentations
2. Logical-mathematical (number/reasoning smart)	Problem-solving, reasoning, patterns, numbers	Puzzles, coding, case studies, experiments, and real-world math applications
3. Spatial (picture smart)	Visualisation, design, drawing, spatial reasoning	Mind maps, infographics, posters, 3D models, and design software projects
4. Bodily-kinesthetic (body smart)	Movement, coordination, hands-on activities	Drama, dance, sports, lab experiments, simulations, and building prototypes
5. Musical (music smart)	Rhythm, sound, tones, music creation	Learning songs, composing rhymes about lessons, and using background music for memorisation
6. Interpersonal (people smart)	Empathy, teamwork, leadership, and communication	Group projects, peer teaching, debates, role-plays, and mentoring activities
7. Intrapersonal (self smart)	Self-awareness, reflection, and independent work	Journals, self-assessment, mindfulness tasks, personal goal setting, and independent study
8. Naturalistic (nature smart)	Connection with nature, ecosystems, and classification	Nature walks, field trips, gardening, environmental projects, and using real-world ecological data

Gardner’s Multiple Intelligences Theory (MIT) advocates for diverse teaching methods—such as verbal, mathematical, visual, kinesthetic, musical, collaborative, reflective, and nature-based strategies—to cater to various learner strengths. However, the theory faces critiques regarding its empirical support and scientific validity; intelligence is complex and challenging to measure independently (Waterhouse, 2006). Cognitive psychology often favors a general intelligence factor (g) rather than distinct intelligences (Neisser et al., 1996). Additionally, the intelligences identified by Gardner may overlap, challenging the idea of their independence (Visser et al., 2006). In practice, these intelligences often work together; for example, writing an essay requires linguistic, logical, and intrapersonal skills. Implementing MIT in education can be challenging. While the framework is appealing, designing lessons that address all intelligences can be time-consuming and resource-intensive (Klein, 1997). Educators may also rely on familiar strategies, limiting the theory’s effectiveness. Moreover, the confusion between multiple intelligences and learning styles can lead to misapplication in schools, despite Gardner’s (1995) clarification of their distinct nature.

### Theoretical model 4: adoption of the classical conditioning learning theory

Learning is fundamentally a process of acquiring new knowledge, behaviors, attitudes, and ideas (Pavlov, 1913; Rehman et al., 2025). This acquisition can occur both consciously and unconsciously (Eelen, 2018) and often involves associations made through experiences (Hall, 2022; Watson and Rayner, 1920). Classical conditioning, also referred to as associative learning, Pavlovian conditioning, or respondent conditioning, represents an unconscious learning process wherein a conditioned response is automatically linked to a specific stimulus (Amd et al., 2019; Totani et al., 2019; Watson, 1924). Pavlov’s ground-breaking research, especially his experiments with dogs, illustrated the principles underlying classical conditioning. He showed that when a neutral stimulus is consistently paired with an unconditioned stimulus, it can provoke a conditioned response, thereby exemplifying the associative learning process (Watson and Rayner, 1920).

The implications of classical conditioning theory are fundamentally significant in the realm of behavioral psychology, shedding light on how environmental factors can profoundly influence and alter human behavior and attitudes. This body of research underscores the concept of behavior as a malleable construct, one that can be modified through appropriate stimuli in the learning environment (Pavlov, 1902; Watson, 1913). For example, a student might develop a lifelong dislike for a subject if they have faced humiliation or punitive measures from a teacher in that context. The interplay of these emotional associations illustrates the critical nature of creating a supportive and positive atmosphere in educational settings, where students can thrive both academically and emotionally.

#### Practical implementation of classical conditioning learning theory

The table below offers some practical ways that the classical conditioning theory can be used in the classroom.

**Training tool: classical conditioning in education**

**Objective**

-  To understand how classical conditioning principles can enhance academic achievement by shaping learners’ behaviors and attitudes towards learning.

**Overview of classical conditioning in education**

1. Establishing positive associations:

-  Pair academic activities with positive stimuli (e.g., praise, rewards, enjoyable learning experiences) to foster a positive learning environment.

2. Reducing anxiety

-  Help learners overcome anxiety related to specific subjects by combining exposure to those subjects with positive experiences. For instance, assist learners who struggle with public speaking by gradually introducing them to it in a supportive setting.

3. Developing positive attitudes

-  Consistently link learning with positive outcomes to help learners develop more favorable attitudes towards education.

Creating routines

-  Establish classroom routines (e.g., starting class with a specific activity or ending with a fun game) that promote positive learning experiences, making the classroom environment more predictable and less stressful.

**Figure 4:**
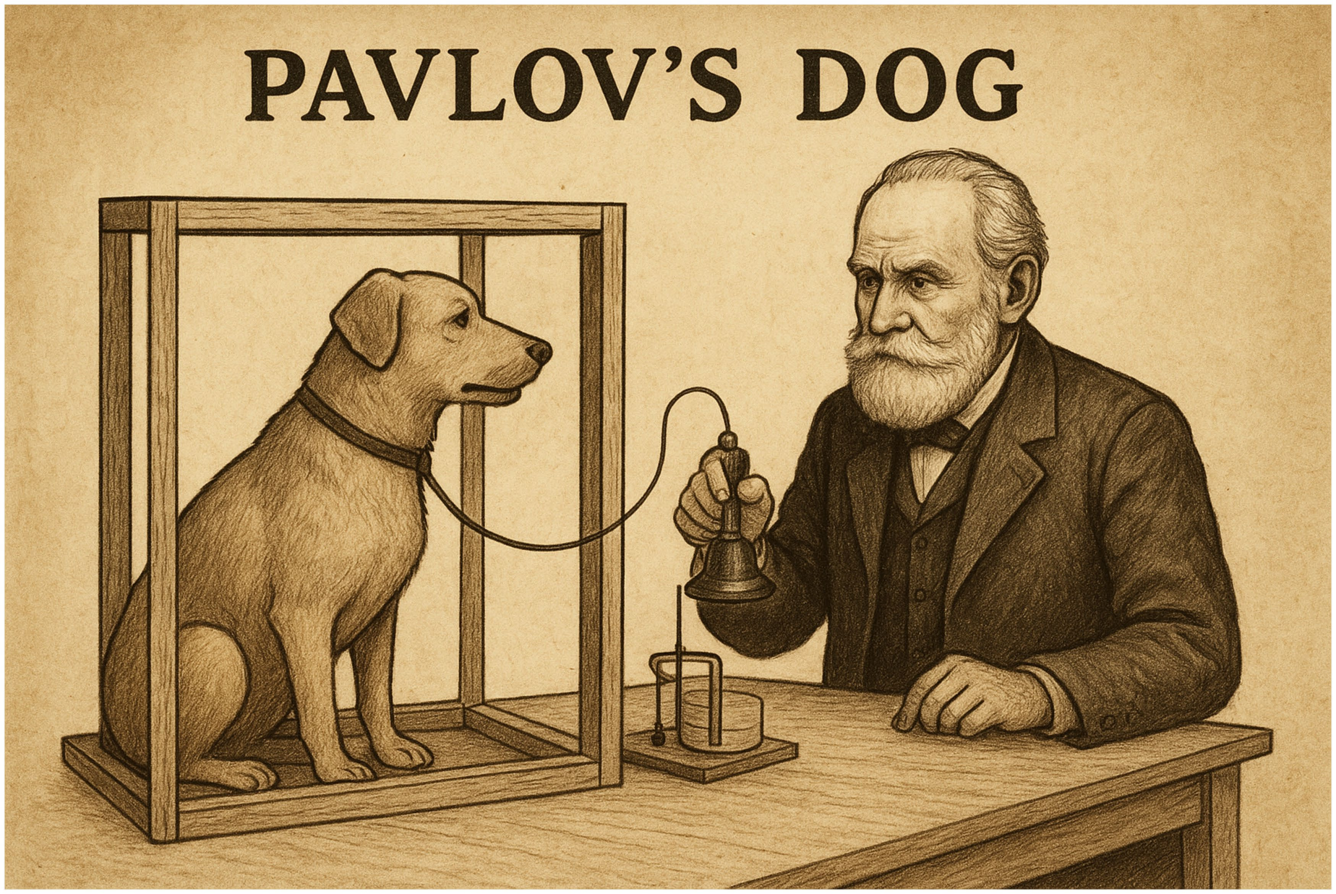
Pavlov’s dog experiment is a classic example of conditioning.


**Three stages of conditioning (see** **Figure 4****)**

**Stage 1: before conditioning:**

In this stage, the unconditioned stimulus (UCS) produces an unconditioned response (UCR) in an organism. In basic terms, this means that a stimulus in the environment has produced a behavior/response that is unlearned (i.e., unconditioned) and, therefore, is a natural response that has not been taught. In this respect, no new behavior has been learned yet.

For example, a stomach virus (UCS) would produce a response of nausea (UCR). In another example, a perfume (UCS) could create a response of happiness or desire (UCR).

This stage also involves another stimulus that has no effect on a person and is called the neutral stimulus (NS). The NS could be a person, object, place, etc.

The neutral stimulus in classical conditioning does not produce a response until it is paired with the unconditioned stimulus.

**Stage 2: during conditioning:**

During this stage, a stimulus which produces no response (i.e., neutral) is associated with the unconditioned stimulus, at which point it now becomes known as the conditioned stimulus (CS).

For example, a stomach virus (UCS) might be associated with eating a certain food, such as chocolate (CS). Also, perfume (UCS) might be associated with a specific person (CS).

For classical conditioning to be effective, the conditioned stimulus should occur before the unconditioned stimulus, rather than after it, or during the same time.

Thus, the conditioned stimulus acts as a type of signal or cue for the unconditioned stimulus.

In some cases, conditioning may take place if the NS occurs after the UCS (backwards conditioning), but this normally disappears quite quickly.

**Stage 3: after conditioning:**

The conditioned stimulus (CS) has been associated with the unconditioned stimulus (UCS) to create a new conditioned response (CR).

For example, a person (CS) who has been associated with nice perfume (UCS) is now found attractive (CR). Also, chocolate (CS), which was eaten before a person was sick with a virus (UCS), now produces a response of nausea (CR).

**Practical examples in the classroom**

***-  Behavioral conditioning***

A teacher places gold stars on the board when learners are quiet and attentive. Over time, learners begin to exhibit quiet and attentive behavior whenever the teacher approaches the chalkboard. This behavior can be explained through classical conditioning:

1. Conditioned Stimulus (CS): Teacher approaching the chalkboard
2. Unconditioned Stimulus (US): Receiving a gold star
3. Conditioned Response (CR): Becoming quiet and attentive

***-  Positive reinforcement***

  -  Praise a student’s efforts on a math problem to create a positive association with the task of working on math. Over time, the student may begin to look forward to math due to the positive feedback received.

***-  Reducing test anxiety***

  -  Encourage learners to visualize a relaxing scene or listen to calming music before tests. This can help them associate relaxation with the testing situation, thus reducing anxiety.

***-  Creating a positive learning environment***

  -  Use a specific song or activity to signal the start of class, helping to establish a positive association with the beginning of the school day.

**Key concepts in classical conditioning**

1. Unconditioned Stimulus (US): A stimulus that naturally elicits a response (e.g., food for a dog).
2. Unconditioned Response (UR): The natural response to the US (e.g., salivation in response to food).
3. Conditioned Stimulus (CS): A neutral stimulus that, through repeated pairings with the US, begins to elicit a response (e.g., a bell sound paired with food).
4. Conditioned Response (CR): The learned response to the CS (e.g., salivation in response to the bell sound).

**Conclusion**

By understanding and applying the principles of classical conditioning, educators can create an environment that not only supports student success but also fosters a positive attitude towards learning. Incorporating these strategies into everyday teaching can significantly enhance learners’ academic experiences.

Through a comprehensive review of existing literature, four distinct theoretical models were identified that can significantly benefit educators. These theoretical models aim to deepen the learning experience by strategically tapping into the EI and CI of students. By leveraging these resources, educators can create a more engaging and supportive learning environment that fosters both emotional development and cognitive growth among learners. The classical conditioning theory has its limitations such as its inability to explain complex human behaviors like reasoning and memory, its disregard for internal cognitive processes, its failure to account for individual differences and free will, and its limited ability to predict behavior in the real world. The theory’s focus on observable actions and its simplistic view of learning also fail to capture the nuances of complex human learning (Brackbill et al., 1967; Hardner et al., 2020; Mackintosh, 1978).

All these theoretical models are intended for both pre-service teacher education and in-service professional development to cater for those still training to become educators/teachers and those who qualified without these valuable models.

## Discussion

This conceptual review aimed to explore ways in which educators worldwide can be trained to be emotionally competent enough to harness learners’ different intelligences for enhanced academic performance and achievement. Multiple intelligences, such as SI, EI, CI, and PI, were identified through the Integrated Human Intelligence Model for Educators, the first theoretical model specifically designed for educators. Various scholars and researchers concluded that a combination of these intelligences, PI, CI, EI, and SI (see Figure 1), leads to well-equipped and developed educators, which ultimately leads to improved academic performance (Grass et al., 2017; Liang et al., 2020; Shi and Qu, 2022). Thus, these core components must be taken into consideration by education and training systems for educators to produce an ideal teacher (Bakir, 2024; Däderman et al., 2013; Farah-Franco et al., 2025; Liu et al., 2021; Lozano-Blasco et al., 2022; Nachiappan et al., 2014; Real-Pérez et al., 2022; Zhou et al., 2024). This implies that this theoretical model or approach to teaching can and will be useful for educators.

Secondly, the Integrated Academic Success Model and VARK Model were identified as other potentially useful theoretical models for educators. This model combines two sensory intelligences, EI and CI, with the Visual, Auditory, Reading/Writing, and Kinesthetic (VARK) learning styles to predict academic success for learners. Emotional intelligence (EI) plays a pivotal role in navigating life’s challenges and has significant implications for academic performance (El-Saftawy et al., 2024). Early development of EI is crucial, and educators are responsible for fostering it through meaningful classroom interactions (Leasa et al., 2017; Hegarty and Angelidis, 2015). Salovey and Mayer (1990) define emotional intelligence as the capacity to understand and regulate emotions, positing that individuals with strong EI are better positioned to learn effectively, irrespective of their learning style (Singh et al., 2008). Notably, the VARK model highlights the importance of sensory modalities in learning, suggesting that learners are likely to process information through emotion, thereby enhancing academic performance (Leasa et al., 2017). Additionally, cognitive intelligence (CI) should be considered alongside EI to bolster understanding of learning styles and their impact on academic success. A high CI indicates an individual’s problem-solving abilities and critical thinking skills (Liu et al., 2021; Stadler et al., 2016; Shi and Qu, 2021, 2022). Both learning styles and CI significantly contribute to intrinsic motivation, facilitating academic success (Kolb, 2005; Li and Bates, 2020). CI serves as a foundational element for effective cognitive learning, equipping learners with essential skills to assimilate and recall information (Demetriou and Spanoudis, 2017; Ronthy, 2014; Zohar and Marshall, 2004).

The third identified theoretical model for educators is a thorough understanding and application of Gardner’s (1983) multiple intelligences theory (MIT). Gardner’s theory proposes that intelligence is not a single ability but a collection of distinct types. Understanding diverse learning styles and intelligences is essential for effective teaching. Felder and Brent (2005) emphasize that recognizing these differences helps educators engage a wider array of learners. By categorizing learners according to various intelligences, educational teams can implement strategies that benefit all learners, not just those strong in math and language (Sener and Cokcaliskan, 2018). Learning styles, encompassing experiential, behavioral, and cognitive traits, reflect how individuals interact with learning environments. Some learners excel with theories, while others prefer active learning or visual aids (Felder and Brent, 2005). An effective approach balances these styles, promoting adaptability in learners. Gardner’s theory highlights the need for learners to understand both their learning style and dominant intelligence for optimal learning (Sener and Cokcaliskan, 2018). Educators should also attempt to understand learners’ most dominant learning styles for specific subjects so they can apply the appropriate learning style(s). Applying this theory can significantly enhance student engagement and foster critical thinking (Calik and Birgili, 2013). Ultimately, it encourages educators to diversify teaching strategies and move beyond traditional methods (Stanford et al., 2003).

The last theoretical model for educators identified in this study was the adoption of classical conditioning learning theory in the classroom. Pavlov (1902) and Watson (1913) examined critical aspects of classical conditioning and concluded that human behavior and attitudes are significantly shaped by environmental stimuli. Notably, they found that the effectiveness of learning acquisition during initial stages is influenced by the visibility of the external stimulus and the timing of the neutral stimulus relative to the unconditioned stimulus (Pavlov, 1902; Rehman et al., 2025). These insights emphasize the importance of educators to create engaging and rewarding learning experiences. Within the educational context, while the implications of classical conditioning may not be as pronounced as those of operant conditioning, it remains essential for educators to cultivate positive emotional associations with learning experiences (Amd et al., 2019; Totani et al., 2019; Watson and Rayner, 1920). An adverse emotional association, such as fear stemming from bullying, can lead to detrimental outcomes, including the development of school phobia. For example, a student who is victimized at school may come to perceive the environment as threatening, thereby fostering aversion to the learning space.

### Limitations and future directions

The current study employs a conceptual review format to investigate the interplay between emotional intelligence (EI) and classroom interactions (CI) across multiple learning modalities. This approach relies on extant theoretical frameworks to delineate the constructs and formulate conclusions. However, this reliance introduces certain limitations, as the theoretical models utilized are often accompanied by inherent shortcomings that could potentially be addressed through empirical evidence.

As a conceptual paper, it is subject to additional constraints, such as the absence of empirical data, the oversimplification of complex realities, and the challenges associated with synthesizing a diverse array of literature. Additionally, potential theoretical gaps and the subjective nature of evaluating conceptual arguments further complicate the analysis, resulting in an increased risk of rejection due to insufficient empirical support for the claims made. Moreover, distinguishing between established theoretical frameworks and novel conceptual ideas can be problematic, which may obscure the paper’s contributions.

Nonetheless, it is crucial to acknowledge that both EI and CI have been extensively researched in relation to academic success. Integrating these concepts with established models, such as the VARK model (Visual, Auditory, Reading/Writing, Kinesthetic), Multiple Intelligences Theory (MIT), and classical conditioning, appears to be a sound methodological approach. Therefore, it is recommended that future research adopt an empirical methodology to investigate the perspectives of learners and educators regarding effective classroom interactions. Furthermore, subsequent studies should explore preferred strategies for enhancing these interactions through the lenses of multiple intelligences and diverse learning styles, thereby fostering a more nuanced understanding of the dynamics within educational settings.

## Conclusion

In conclusion, the development of flexible teaching approaches that cater to a diverse range of learning preferences is crucial in the contemporary educational landscape. By integrating a variety of instructional methods and resources, educators can cultivate a dynamic and inclusive environment that empowers all learners. The emphasis on specialized training for educators significantly enhances their ability to implement effective emotional and intellectual strategies, thereby contributing to improved academic performance and increased student engagement. Additionally, adopting a student-centred approach fosters learner autonomy and facilitates self-directed exploration, both of which are essential for success in the modern classroom. It is imperative that educational institutions and curriculum developers prioritize these methodologies to ensure that every learner is afforded the opportunity to thrive.
